# Conditioned Media of Choroid Plexus Epithelium Cells Attenuates High Pi-Induced Calcification of MOVAS Cells by Inhibiting ROS-Mediated Signal Pathways

**DOI:** 10.3389/fphys.2021.607739

**Published:** 2021-02-05

**Authors:** Xin Hui, Mei Wang, Lijun Zhang, Ji Liu, Mengen Wang, Weiwei Hu, Tongtong Zhang, Shijun Zhao, Shangyong Geng, Xianjun Wang, Zuncheng Zheng

**Affiliations:** ^1^Department of Neurology, Linyi People’s Hospital, Linyi, China; ^2^Department of Ultrasonography, Taian City Central Hospital, Tai’an, China; ^3^Department of Neurology, Baotou Central Hospital, Baotou, China; ^4^Department of Rehabilitation, Tai’an City Central Hospital, Tai’an, China

**Keywords:** vascular calcification, choroid plexus epithelium cells, ROS, DNA damage, MAPKs and PI3K/AKT

## Abstract

Vascular calcification was an independent risk of cardiovascular and cerebrovascular diseases (CCDs). Studies reported that conditioned media of choroid plexus epithelium cells (CPECs-CM) showed potential neuroprotective effects. However, the protective effect of CPECs-CM against vascular calcification (VC) has not been reported yet. Herein, high phosphate (HPi)–induced calcification model in mouse aortic vascular smooth muscle cells (MOVAS) was established, and the protective effects and underlying mechanism of CPECs-CM against HPi-induced calcification were explored. The results indicated that CPEC cells were successfully isolated and cultured, and CPECs-CM co-treatment significantly inhibited HPi-induced calcification of MOVAS cells through blocking alkaline phosphatase activity and expression. CPECs-CM co-treatment also suppressed reactive oxide species–mediated DNA damage in HPi-treated MOVAS cells. Moreover, dysfunction of MAPKs and PI3K/AKT pathways both contributed to HPi-induced calcification of MOVAS cells, and CPECs-CM co-treatment attenuated HPi-induced calcification by normalizing MAPKs and PI3K/AKT expression. Taken together, our findings provide evidence that CPECs-CM had the potential to inhibit vascular calcification with potent application in chemoprevention and chemotherapy of human CCD.

## Introduction

Vascular calcification was accepted as a common pathological feature of atherosclerosis, diabetes, hypertension, kidney disease, and aging. Vascular calcification was related to the occurrence, progression, and prognosis of ischemic stroke, which is one of the most important diseases threatening global human health with the characteristic of high morbidity, high fatality rate, high disability rate, and high recurrence rate (Kao et al., 2015; Gocmen et al., 2018; Kong et al., 2019).

Abnormal deposition of the mineral calcium phosphate on blood vessel wall is an active biological process, and was regulated by multiple factors and pathways, which is similar to osteogenesis. A large number of cytokines and growth factors, including pro-inflammatory cytokines IL-6 and tumor necrosis factor-α (TNFα), osteoprotegerin, sclerostin, matrix gamma-carboxyglutamic acid-rich (GLA) protein (MGP), and fibroblast growth factor (FGF)-23, were all involved in the vascular calcification process (Durham et al., 2018). Vascular calcification usually occurs in the vascular wall, and was associated with aging, diabetes, and kidney disease (Disthabanchong and Srisuwarn, 2019; Proudfoot, 2019). Increased evidence suggested that the generation of apoptotic corpuscles by apoptosis of vascular smooth muscle cells can act as nucleating structures and initiate vascular calcification (Wang et al., 2018). Furthermore, oxidative stress activates endoplasmic reticulum stress in vascular smooth muscle cells, leading to increased apoptosis and vascular calcification (Ducy et al., 1997; Liu et al., 2014). Previous studies reported that runt-related transcription factor 2 (RUNX2) as an osteogenic marker could regulate osteoblasts and chondrocyte differentiation by inducing alkaline phosphatase (ALP) activity and the expression of bone matrix protein genes *in vitro* (Inada et al., 1999; Lian et al., 2004). The exogenous hydrogen peroxide can induce calcification in vascular smooth muscle cells via regulating Runx2 and PI3K/AKT signaling (Mody et al., 2001; Byon et al., 2008). Moreover, activation of p38 MAPK also could induce bone cell differentiation and bone formation through interacting with Runx2 in response to oxidative stress (Yang et al., 2018).

Choroid plexus epithelial cells (CPECs) are the main components of choroid plexus, which plays a key role in maintaining central nervous system (CNS) homeostasis, and they participate in the formation of the blood–brain barrier. CPECs exhibit multiple functions, such as exchange of ions, transport of nutrients and metabolites, and secretion of cerebrospinal fluid (about 70–80%) and other substances. CPECs also play a positive role in post-traumatic repair *in vitro* (Emerich et al., 2004). Aliaghaei et al. (2014) reported CPECs could secrete many neurotrophic factors, nourish the nerves, and promote the axon extension of the cells. In addition, CPECs show potential to inhibit the generation of free radicals by activating the Nrf2/antioxidant response elements (ARE) pathway (Aliaghaei et al., 2014). However, the protective effect and mechanism of CPECs against calcification of MOVAS cells have not been investigated yet.

## Materials and Methods

### Materials

2’,7’-Dichlorofluorescein diacetate (DCFH-DA), bicinchoninic acid (BCA) kit, ALP staining kit, and ALP activity kit were bought from Beyotime (Shanghai, China). DMEM-F12, fetal bovine serum (FBS), and penicillin–streptomycin were obtained from Invitrogen (Carlsbad, CA, United States). U0126 (ERK inhibitor) and main antibodies, such as p-ATM (#9607), p-p53 (#9284), p-H_2_A (#9718S), p-JNK (#9255), p-p38 (#4511), JNK (#9252), p38 (#2383), p-ERK (#4370), ERK (#4695), p-AKT (#4060), and AKT (#9272), were obtained from Cell Signaling Technology (CST, Beverly, MA, United States). All agents used were of high-performance liquid chromatography (HPLC) grade.

### Isolation, Culture, and Identification of Choroid Plexus Epithelium Cells

SD rats were fed and newborn SD rats (2018-0019) were administered with isoflurane for deep anesthesia. Rat brains were removed and washed with PBS, and sections of rat brain were stained with H&E for histological observation. Briefly, rat brains were fixed in paraformaldehyde (4%), embedded in paraffin, and cut into 4-μm slices. Then, brain tissue was given gradient alcohol dehydration and stained with H&E, respectively. The morphology of choroid plexus was observed by a light microscope. Choroid plexus tissue was isolated from the ventricle under aseptic procedures. Tissues were cut into pieces and centrifuged, and CPECs were cultured with DMEM/F12 medium with 10% fetal bovine serum (FBS). Conditioned media of CPECs (CPECs-CM) was collected and stored at −20°C for further use. Transthyretin (TTR) as a marker of CPECs was employed to identify the purity. Briefly, CPECs seeded in a 6-well plate were fixed with 4% formaldehyde. Then, cells were incubated with TTR primary antibody at 4°C overnight. Then, cells were washed and incubated with FITC-labeled secondary antibody at room temperature for 2 h. Cells were washed and stained with DAPI. TTR expression was imaged under a fluorescent microscope.

### Cell Culture and *in vitro* Calcification

Mouse aortic vascular smooth muscle cells (MOVAS) were obtained from American Type Culture Collection (ATCC, Manassas, VA, United States) and cultured in DMEM with 10% FBS and 1% penicillin and streptomycin at 37°C in a 5% CO_2_ incubator. For calcification treatment, cells were cultured with calcifying medium (β-glycerophosphate, pH 7.4). Briefly, MOVAS cells were cultured with CPECs-CM and/or co-treated with 3 mM high phosphate for 14 days. MOVAS cell calcification was detected by other methods.

### Alizarin Red Staining

MOVAS cells were cultured with CPECs-CM and/or co-incubated with 3 mM high phosphate for 14 days. After incubation, cells were fixed in 4% formaldehyde for 10 min and incubated at room temperature with 2% alizarin red (pH 4.2) for 5 min. Excess dye was washed and calcification status was imaged by an inverted phase contrast microscope. Alizarin red dye was then eluted with 10% formic acid and quantified by a microplate reader at 420 nm to record the absorbance of eluted alizarin red. Calcium content was also quantified by calcium detecting kit. Briefly, MOVAS cells, after treatment with CPECs-CM and/or 3 mM high phosphate, were lysed by RIPA solution, and total protein was extracted and quantified by BCA protein assay kit. Then, protein was added into a 96-well plate, and was added buffer and standard solution according to the instruction. Calcium content was quantified by O-cresolphthalein complexone method.

### ALP Staining and Measurement of ALP Activity

Alkaline phosphatase staining and ALP activity were performed as previously reported (Nakano-Kurimoto et al., 2009). Briefly, MOVAS cells were cultured with CPECs-CM and/or co-incubated with 3 mM high phosphate for 14 days. After incubation, cells were fixed in 4% formaldehyde for 10 min, and ALP staining was conducted with an ALP staining kit. ALP activity was measured by an ALP activity kit. Briefly, MOVAS cells, after treatment with CPECs-CM and/or 3 mM high phosphate, were lysed by RIPA solution, and total protein was extracted and quantified by BCA protein assay kit. Then, total protein (40 μg) was added to 180 μl p-NPP substrate and incubated for 20 min at 37°C. ALP activity was measured at 405 nm and calculated as nanomoles per milliliter *p*-nitrophenol converted per microgram of protein per minute.

### Detection of ROS Generation and Superoxide Anion

Intracellular reactive oxygen species (ROS) and superoxide anion were detected by DCFH-DA and DHE probes, respectively. Briefly, MOVAS cells were treated with 3 mM HPi for 0–90 min. After treatment, cells were washed with PBS and labeled with 10 μM DCFH-DA or DHE probes for 15 min at darkness. Then, ROS generation (green fluorescence) and superoxide anion (red fluorescence) were both imaged under a fluorescent microscope. Meanwhile, intracellular ROS and superoxide anion were also quantified by a microreader (SpectraMax, M5). All data was expressed as percentage of control group (as 100%).

### Western Blotting

MOVAS cells were cultured with CPECs-CM and/or co-incubated with 3 mM high phosphate for 14 days. After incubation, MOVAS cells were incubated with the lysis buffer containing PMSF for 30 min, and then the cells were moved to a 1.5-ml centrifuge tube to centrifuge at 12,000 × *g* under 4°C for 5 min. The total proteins were extracted from supernatant fluid, and the protein concentration was detected by BCA method. Before transferring to nitrocellulose membrane, equal amounts of protein were separated with 10% sodium dodecyl sulfate-polyacrylamide gel (SDS-PAGE). Then, the membranes were blocked with 5% (w/v) non-fat milk for 1 h and incubated with the specific primary antibody overnight under 4°C. The membranes were incubated with secondary antibody for 1 h at room temperature. Protein expression was observed with enhanced chemiluminescence detection system. β-Actin was set to be standardized as a reference.

### Statistical Analysis

All results were from at least three independent experiments and are expressed as mean ± SE. Statistical analyses were performed by the standard of one-way ANOVA by SPSS 22.0 software. Also, two-tailed Student’s *t*-test was used to evaluate the differences between two groups. Bars with different letters indicate the significance at *P* < 0.05 level.

## Results

### Isolation, Culture, and Identification of CPECs

**FIGURE 1:**
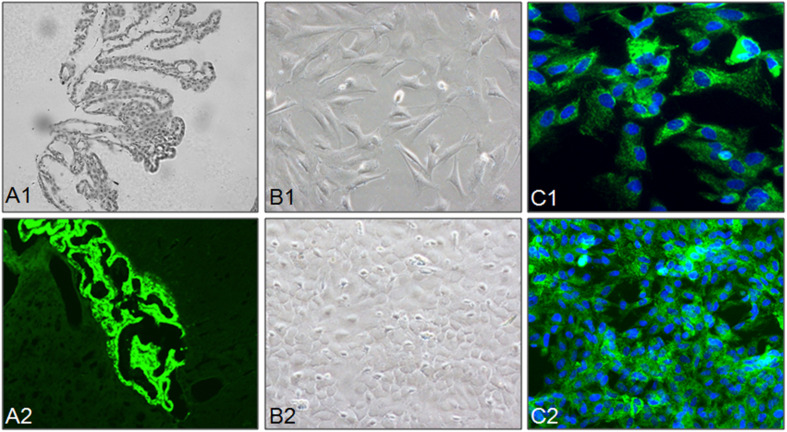
Isolation, culture, and identification of CPECs. **(A1,A2)** Identification of choroid plexus in rat brain. **(B1,B2)** CPECs were isolated and cultured with cultured with DMEM/F12 medium. The morphology of CPECs (**B1**: low concentration, **B2**: high concentration) was observed by phase microscope. Morphology of isolated CPECs. **(C1,C2)** Identification of isolated CPECs. CPECs were labeled by immunofluorescent staining using a transthyretin (TTR) antibody, a CPEC marker.

First, choroid plexus was observed by H&E staining, and the result indicated that choroid plexus showed a bunch-of-grapes appearance (Figure 1A1). Second, CPECs showed strong positive TTR expression (Figure 1A2), a marker for CPECs (Herbert et al., 1986). CPECs were isolated from rat brains and cultured in DMEM-F12 medium, and the cells showed a triangular diamond shape (Figure 1B1). After 1-week culture, CPECs showed a high concentration growth (Figure 1B2). TTR staining indicated that CPECs with low and high cell concentration both showed strong positive expression of TTR (Figures 1 C1,C2). These results suggested that CPECs were successfully isolated and cultured and showed high purity.

### CPECs-CM Attenuates HPi-Induced Calcification of MOVAS Cells

**FIGURE 2:**
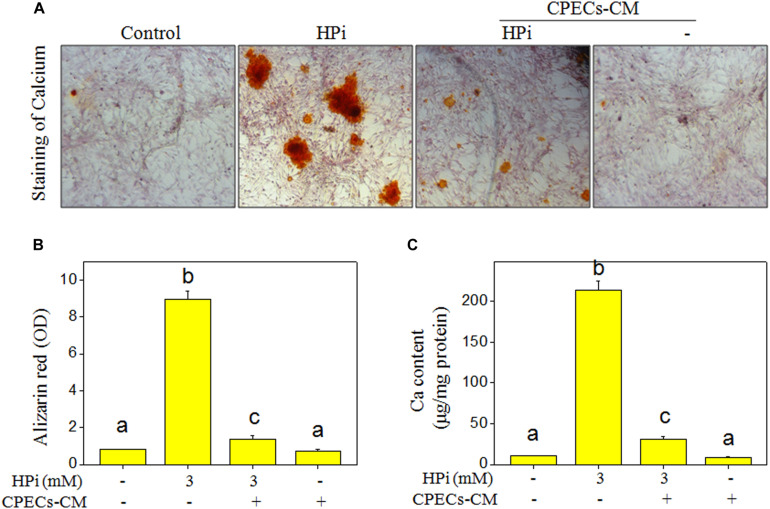
CPECs-CM attenuates high Pi-induced calcification of MOVAS cells. **(A)** CPECs-CM inhibited high Pi-induced calcification of MOVAS cells. MOVAS cells seeded in a 6-well plate were cultured with CPECs-CM and treated with 3 mM high Pi for 14 days. Calcium nodules were stained by alizarin red. **(B)** Absorbance of alizarin red. Alizarin red dye was eluted with 10% formic acid and quantified by a microplate reader at 420 nm. **(C)** Measurement of calcium content. Protein was quantified by BCA kit, and O-cresolphthalein complexone method was used to examine calcium content as previously described. Ca content was expressed as micrograms per milligram of protein. All data were obtained from three independent experiments. Bars with different letters indicate significance at *P* < 0.05 level.

Previous studies have demonstrated that smooth muscle cells exposed to high Pi *in vitro* will be calcified and exhibit osteochondrogenic phenotype (Jono et al., 2000; Sugitani et al., 2003). Therefore, MOVAS calcification exposed to 3 mM HPi was ascertained by alizarin red staining. As shown in Figure 2A, HPi treatment induced significant calcification of MOVAS cells, as convinced by the increased calcium nodules. However, co-treatment with CPECs-CM effectively attenuated HPi-induced calcification of MOVAS cells. CPECs-CM treatment alone caused no significant calcification of MOVAS cells. To further determine the protective effect of CEPCs-CM, the absorbance of eluted alizarin red was measured and calcium content was quantified, and the results indicated that CPECs-CM co-treatment completely inhibited the absorbance of eluted alizarin red (Figure 2B) and calcium content in HPi-treated MOVAS cells (Figure 2C). Taken together, these results suggested that CEPCs-CM had the potential to attenuate HPi-induced calcification of MOVAS cells.

### CPECs-CM Suppressed HPi-Induced Osteogenic Differentiation

**FIGURE 3:**
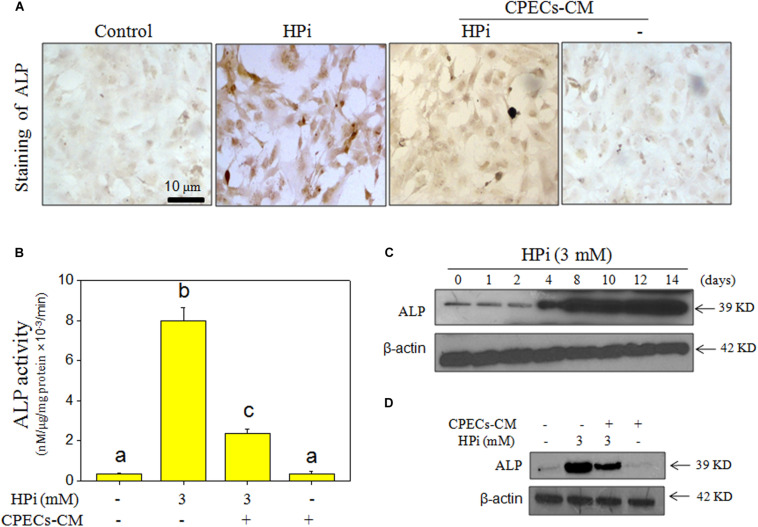
CPECs-CM suppressed high Pi-induced ALP activity and expression. **(A)** ALP staining of MOVAS cells. MOVAS cells seeded in a 6-well plate were cultured with CPECs-CM and treated with 3 mM high Pi for 14 days. ALP staining was conducted by an ALP staining kit. **(B)** Measurement of ALP activity. Total protein was extracted and quantified by BCA kit. ALP activity was examined by an ALP activity kit according to the manufacturer’s instructions. **(C,D)** ALP expression was detected by western blotting method. All data were obtained from three independent experiments. Bars with different letters indicate significance at *P* < 0.05 level.

Alkaline phosphatase was a marker of osteogenic differentiation, and knockdown of ALP significantly attenuates calcification in senescent vascular smooth muscle cells (VSMCs) (Nakano-Kurimoto et al., 2009). Therefore, ALP activity and ALP expression in HPi-treated MOVAS cells were both detected. As shown in Figure 3A, ALP staining result suggested that MOVAS cells after HPi treatment showed enhanced ALP staining. HPi treatment also caused a significant increase in ALP activity (Figure 3B). The time-dependent increase of ALP expression further confirmed HPi-induced osteogenic differentiation of MOVAS cells (Figure 3C). However, CPECs-CM co-treatment significantly suppressed HPi-induced ALP staining, ALP activity, and ALP expression. MOVAS cells exposed to CPECs-CM alone showed no effects on ALP staining, ALP activity, and ALP expression (Figure 3D). The quantitative analysis of ALP expression further confirmed this conclusion (Supplementary Figure S1). Moreover, runt-related transcription factor 2 (RUNX2) as another osteogenic marker was also detected, and the result further confirmed that CPECs-CM suppressed HPi-induced osteogenic differentiation of MOVAS cells (Supplementary Figure S2). Taken together, these results indicated that CPECs-CM had the potential to suppress HPi-induced osteogenic differentiation of MOVAS cells.

### CPECs-CM Blocks HPi-Induced DNA Damage

**FIGURE 4:**
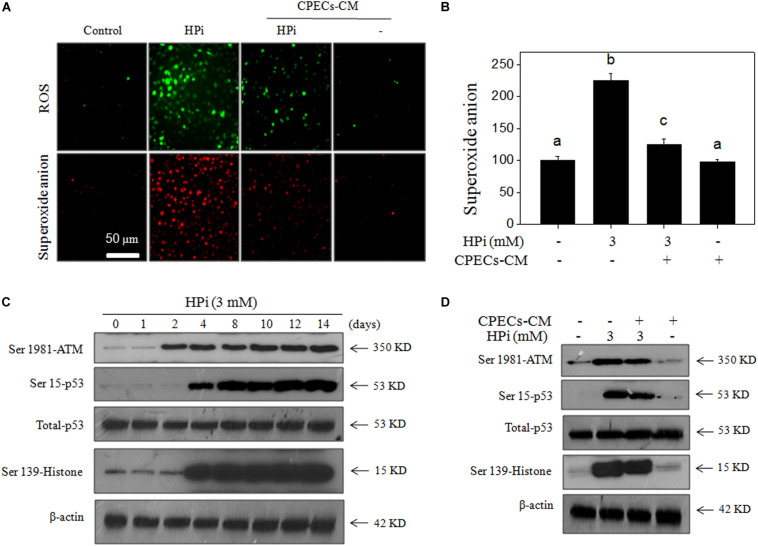
CPECs-CM prevents high Pi-induced ROS generation and DNA damage. **(A)** Real-time imaging of ROS generation and superoxide anion. MOVAS cells were seeded in a 6-well plate and pre-loaded with DCFH-DA or DHE probes for 15 min in the dark. Then cells were washed with PBS and treated with 3 mM high Pi or/and CPECs-CM for 90 min. The ROS generation (green fluorescence) and superoxide anion (red fluorescence) were imaged by a fluorescent microscope. **(B)** Quantitative analysis of superoxide anion. Intracellular superoxide anion generation was quantified by a microreader. **(C)** Real-time DNA damage. MOVAS cells were seeded in a 9-cm plate and treated with 3 mM high Pi for 1, 2, 4, 8, 10, 12, and 14 days. **(D)** CPECs-CM inhibited high Pi-induced DNA damage. MOVAS cells seeded in a 9-cm plate were cultured with CPECs-CM and treated with 3 mM high Pi for 14 days. Protein expression was examined by western blotting. All experiments were done at least three times.

It was reported that ROS-mediated oxidative damage was associated with HPi-induced calcification (Trion and van der Laarse, 2004; Choi et al., 2017). Therefore, HPi-induced accumulation of ROS and superoxide anion, and DNA damage were all detected in MOVAS cells. As shown in Figure 4A, HPi treatment induced significant generation of ROS and superoxide anion in a time-dependent manner, as demonstrated by the increased green and red fluorescence, respectively. The quantitative result of superoxide anion further confirmed this conclusion (Figure 4B). CPECs-CM alone showed no effect on superoxide anion (Figure 4B). In order to further confirm the significant role of HPi-induced ROS, several ROS inhibitors were employed. The results indicated that three thiol-reducing antioxidants, including GSH, NAC, and GSH-er, at 5 mM, effectively inhibited HPi-induced ROS generation. DPIC (inhibitor of xanthine oxidase and NADPH oxidase, 10 μM) also showed significant inhibitory effect on HPi-induced ROS generation (Supplementary Figure S3).

HPi-induced DNA damage was subsequently detected by western blotting method. As shown in Figure 4C, several DNA damaging markers were examined and the results indicated that HPi treatment significantly increased the phosphorylation level of ATM (Ser1981), p53 (Ser15), and histone (Ser 139), indicating that HPi treatment caused DNA damage in MOVAS cells. As expected, CPECs-CM co-treatment markedly blocked HPi-induced DNA damage, as demonstrated by the decreased phosphorylation level of ATM (Ser1981), p53 (Ser15), and histone (Ser 139) (Figure 4D). The quantitative analysis of p-ATM, p-p53, and p-H2A expression further confirmed the conclusion (Supplementary Figure S4). Moreover, CPECs-CM co-treatment also inhibited HPi-induced cytochrome *c* release (Supplementary Figure S5). Taken together, these results suggested that CPECs-CM had the potential to block ROS-induced oxidative damage in HPi-treated MOVAS cells.

### CPECs-CM Normalizes MAPKs and PI3K/AKT Pathways

**FIGURE 5:**
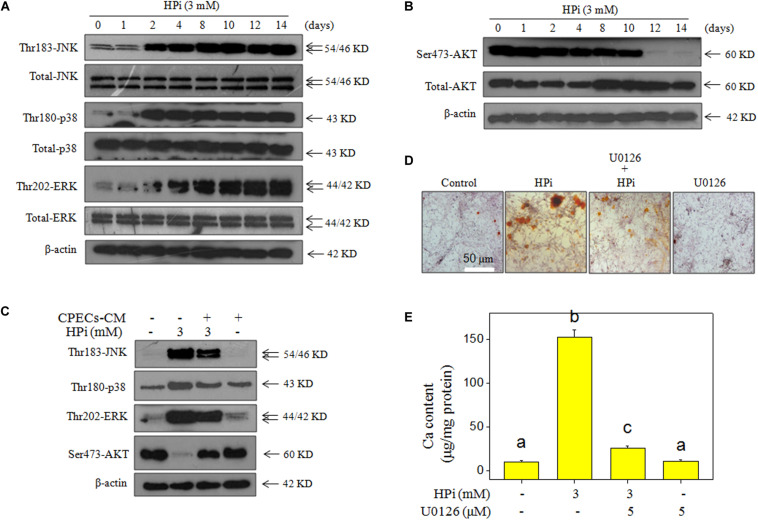
CPECs-CM improves high Pi-induced dysfunction of MAPKs and PI3K/AKT. Time-dependent effect of high Pi on MAPKs **(A)** and PI3K/AKT **(B)** expression. MOVAS cells seeded in a 9-cm plate were treated with 3 mM high Pi for 1, 2, 4, 8, 10, 12, and 14 days. **(C)** CPECs-CM improved the expression of MAPKs and PI3K/AKT pathways in high Pi-treated cells. **(D)** U0126 (an ERK inhibitor) inhibited high Pi-induced calcification of MOVAS cells. MOVAS cells seeded in a 9-well plate were cultured with CPECs-CM and treated with 3 mM high Pi for 14 days. **(E)** Measurement of calcium content. Protein expression was examined by western blotting. All data were obtained from three independent experiments. Bars with different letters indicate significance at *P* < 0.05 level.

MAPKs and PI3K/AKT pathways both contributed to HPi-induced calcification (Byon et al., 2008). To further identify the underlying signal mechanisms by which CPECs-CM inhibit HPi-induced calcification, MAPK and PI3K/AKT members were all examined by western blotting method. As shown in Figure 5A, MOVAS cells exposed to 3 mM high Pi showed a significant increase in phosphorylation level of JNK (Thr183), p38 (Thr180), and ERK (Thr202) in a time-dependent manner. Phosphorylation level of AKT (Ser473) in HPi-treated MOVAS cells displayed a sustained decrease (Figure 5B). HPi treatment alone caused no significant changes of total JNK, p38, and AKT. However, abnormal phosphorylation level of JNK (Thr183), p38 (Thr180), ERK (Thr202), and AKT (Ser473) in HPi-treated MOVAS cells were all effectively normalized after CPECs-CM co-treatment (Figure 5C). Inhibitor of ERK (U0126) significantly inhibited HPi-induced calcification of MOVAS cells (Figure 5D), and the quantitative result of calcium content further confirmed this effect (Figure 5E). Moreover, pre-treatment of cells with another three inhibitors, SB203580 (p38 inhibitor), SP600125 (JNK inhibitor), and LY294002 (AKT inhibitor), further confirmed that dysfunction of MAPKs and PI3K/AKT pathways all contributed to HPi-induced calcification (Supplementary Figure S6). The quantitative analysis of p-JNK, p-p38, p-ERK, and p-AKT expression further confirmed the conclusion (Supplementary Figure S7). Taken together, these results revealed that CEPCs-CM could inhibit HPi-induced calcification of MOVAS cells by normalizing MAPKs and PI3K/AKT expression.

## Discussion

Previous studies have confirmed that CPECs-CM showed prominent neuroprotective effects, and infusion of CPECs had the potential to suppress ischemic damage to the brain (Borlongan et al., 2004; Matsumoto et al., 2010). It was reported that CPECs-CM could attenuate H_2_O_2_-mediated apoptosis in PC12 cells by activating Nrf2/ARE and inhibiting oxidative stress (Aliaghaei et al., 2014), and eventually suppress MOVAS calcification. However, the protective effect and mechanism of CPECs-CM against HPi-induced calcification of MOVAS cells has not been reported.

Phosphorus-induced osteogenic/chondrogenic transformation, differentiation, and calcification are directly associated with ROS in previous studies (Choi et al., 2017; Luong et al., 2018). Production of ROS in the present study was increased in high Pi-induced MOVAS cells. Oxidative stress can induce apoptosis of vascular smooth muscle cells (Gomez et al., 2015), which may promote the occurrence of vascular calcification (Paloian and Giachelli, 2014). Hydrogen peroxide (H_2_O_2_) has been reported to trigger typical DNA damage pathways, including single and double strain breaks (Driessens et al., 2009). ROS participates in redox signal transduction, and causes oxidative damage to cells, such as DNA damage. Furthermore, the reduction of mitochondrial membrane potential (MMP) could lead to production of ROS (Rigoulet et al., 2011; Sosa et al., 2013) and result in releasing of the apoptogenic factors from the mitochondrial intermembrane space to the cytoplasm, which will activate the caspase cascade and eventually trigger cell apoptosis (Choi et al., 2017; Luong et al., 2018).

DNA damage response (DDR) can be activated in response to DNA damage and trigger DNA damage repair (Shiloh and Ziv, 2013). ATR and ATM as major effectors of DDR can be activated firstly in response to oxidizing agents (Kurz et al., 2004). In the present study, exposure of MOVAS cells to HPi showed a significant increase in ATM expression. DNA damage signaling has been identified as key mediators in osteogenic differentiation of VSMCs (Liu et al., 2013). p53 is involved in the repair of DNA damage, and the increased expression of p53 meant occurrence of DNA damage in cells exposed to high Pi (Figure 4B). Phosphorylation of p53 on Ser15, which occurs rapidly after DNA damage, could block ubiquitination and proteasome degradation of p53, which contributed to the overall stability of p53 (Kastan et al., 1991; Wartlick et al., 2013; Borodkina et al., 2014). Activated p53 can trigger DNA damage repair and induce apoptosis of MOVAS cells. In the present study, CPECs-CM co-treatment effectively attenuated HPi-induced p53 expression, indicating that CPECs-CM contained antioxidant agents, which can inhibit ROS-mediated DNA damage.

Oxidative stress has shown to be associated with dysfunction of MAPKs and PI3K/AKT pathways (Liu et al., 2014). Dysfunction of MAPKs and PI3K/AKT pathways contributed to the osteogenic differentiation of vascular smooth muscle cells (Liu et al., 2014). Increasing studies have verified that the vascular calcification will be promoted by apoptosis of VSMCs (Proudfoot et al., 2000; Collett et al., 2007; Clarke et al., 2008), and dysfunction of PI3K/AKT signaling (Downward, 2004). In the present study, CPECs-CM co-treatment significantly improved the phosphorylation level of PI3K/AKT pathways and inhibited MOVAS calcification. MAPK signaling has the ability to promote osteocyte differentiation and bone formation by regulating phosphorylate Runx2 and increasing Runx2 *trans-*activity (Simmons et al., 2004; Greenblatt et al., 2010; Zhou et al., 2014; Yang et al., 2018). P38 as one important member of MAPK signal was involved in regulating the shape of vascular calcification (Zhou et al., 2014; Li et al., 2016, 2017). However, activation of p38 MAPK was effectively inhibited in HPi-treated MOVAS cells. These results revealed that CEPCs-CM could inhibit HPi-induced calcification of MOVAS cells by normalizing MAPKs and PI3K/AKT expression. CPECs can synthesize and secrete numerous neurotrophic factors, such as nerve growth factor (NGF), brain-derived neurotrophic factor (BDNF), neurotrophin 3–4 (NT3–4), vascular endothelial growth factor (VEGF), and fibroblast growth factor 2 (FGF2). We speculated that the conditioned media contained these factors and many antioxidases, which had the potential to inhibit ROS-mediated oxidative damage and attenuate HPi-induced calcification of MOVAS cells.

## Conclusion

**FIGURE 6:**
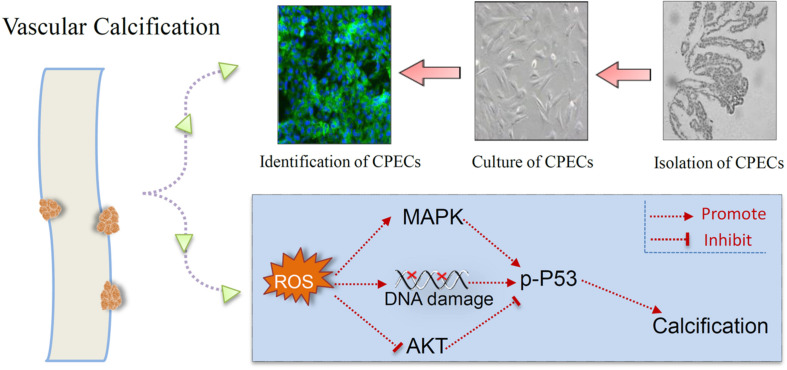
Proposed signal pathways. CPECs were isolated, cultured, and identified. CPECs-CM co-treatment effectively inhibited HPi-induced ROS generation, DNA damage, and dysfunction of MAPKs and PI3K/AKT pathways, and eventually attenuated HPi-induced calcification of MOVAS cells.

In the present study, CPECs were successfully isolated and cultured, and CPECs-CM co-treatment effectively inhibited HPi-induced calcification of MOVAS cells by decreasing ALP activity and expression. CPECs-CM co-treatment also attenuated ROS-mediated DNA damage and normalized MAPKs and PI3K/AKT expression in HPi-treated MOVAS cells (Figure 6). Our findings provide evidence that CPECs-CM had the potential to inhibit vascular calcification with potent application in chemoprevention and chemotherapy of human CCD.

## Data Availability Statement

The original contributions presented in the study are included in the article/Supplementary Material, further inquiries can be directed to the corresponding authors.

## Ethics Statement

The animal study was reviewed and approved by the Taian City Central Hospital Committee for Laboratory Animal Research.

## Author Contributions

XW, ZZ, and SG designed the experiments. XH, MiW, JL, MnW, LZ, WH, TZ, and SZ performed the experiments. XH and LZ wrote the manuscript. All authors analyzed the data, images, and reviewed the article.

## Conflict of Interest

The authors declare that the research was conducted in the absence of any commercial or financial relationships that could be construed as a potential conflict of interest.
