# Supplementary material for: Conditioned Media of Choroid Plexus Epithelium Cells Attenuates High Pi-Induced Calcification of MOVAS Cells by Inhibiting ROS-Mediated Signal Pathways
Source: Front Physiol. 2021 Feb 5;12:607739. doi: 10.3389/fphys.2021.607739 (PMC7892975; doi:10.3389/fphys.2021.607739)
Supplement: Supplementary file 1 [file Table_1.DOC]

**Supporting Information**

**For**

**Conditioned media of** **choroid plexus epithelium cells** **attenuates** **high Pi****-induced calcification of MOVAS cells by inhibiting ROS-mediated signal pathways**

Xin Hui^1‡^, Mei Wang^2‡^, Lijun Zhang^1^, Ji Liu^1^, Mengen Wang^1^, Weiwei Hu^2^, Tongtong Zhang^1^, Shijun Zhao^3^, Shangyong Geng^3*^, Xianjun Wang^1*^, Zuncheng Zheng^4*^

^1^ Department of Neurology, Linyi People’s Hospital, Linyi, 276000, Shandong, China

^2^ Departments of Ultrasonography, Taian City Central Hospital, Taian 271000, Shandong, China

^3^ Department of Neurology, Baotou Central Hospital, Baotou, 014040, Neimenggu, China

^4^ Departments of Rehabilitation, Taian City Central Hospital, Taian 271000, Shandong, China

***Corresponding authors.**

**Xianjun Wang**, Department of Neurology, People’s Hospital of Linyi Affiliated to Shandong First Medical University, Linyi, 276000, Shandong, China/ Email: wangxianjun008@163.com

**Zuncheng Zheng,** Longtan Road, Taian City Central Hospital, Taian, 271000, Shandong, China. Email: zhengzc1965@126.com

**Shangyong Geng**, Department of Neurology, Baotou Central Hospital, Baotou, 014040, Neimenggu, China. Email: sygen1999@163.com

**Figure S1.** **Quantitative analysis of ALP expression.** MOVAS cells seeded in 9-cm plate were treated with 3 mM HPi for 0-14 days, or cells were cultured with CPECs-CM and treated with or without 3 mM high Pi for 14 days. ALP Protein expression was examined by western blotting. Bars with different letters indicate the significance at P<0.05 level.

**Figure S2. CPECs-CM inhibited HPi-induced RUNX2 expression.** MOVAS cells seeded in 9-cm plate were cultured with CPECs-CM and treated with or without 3 mM high Pi for 14 days. Protein expression was examined by western blotting.

**Figure S3. Inhibitory effect of several ROS inhibitors on ROS accumulation.** MOVAS cells seeded in 96-well plate were pre-treated with 5 mM glutathione ethylene ester (GSH-er), 5 mM glutathione (GSH), 5 mM N-acetylcysteine (NAC), 10 μM diphenyleneiodonium chloride (DPIC) and 10 mM DMSO for 2 h and co-treated with 3 mM high phosphate for 90 min. ROS generation was detected by DCFH-DA probe, and quantified by a micro-reader.

**Figure S4.** **Quantitative analysis of p-ATM, p-p53 and p-H2A expression.** MOVAS cells seeded in 9-cm plate were treated with 3 mM HPi for 0-14 days, or cells were cultured with CPECs-CM and treated with or without 3 mM high Pi for 14 days. p-ATM, p-p53 and p-H2A expression were examined by western blotting. Bars with different letters indicate the significance at P<0.05 level.

**Figure S5. CPECs-CM inhibited HPi-induced cytochrome C release.** MOVAS cells seeded in 9-cm plate were cultured with CPECs-CM and treated with or without 3 mM high Pi for 14 days. Cytochrome C expression in cytoplasm was examined by western blotting method. Protein expression was examined by western blotting.

**Figure S6. Inhibitory effects of different kinases inhibitors on HPi-induced calcification of MOVAS cells.** MOVAS cells seeded in 9-cm plate were pre-treated with 5 µM SB203580 (p38 inhibitor), 5 µM SP600125 (JNK inhibitor), or 5 µM LY294002 (AKT inhibitor) before HPi treatment. Protein was quantified by BCA kit, and O-cresolphthalein complexone method was used to examine calcium content as previously described. Ca content was expressed as μg/mg protein. All data were obtained from three independent experiments. Bars with different letters indicate the significance at P<0.05 level.

**Figure S7.** **Quantitative analysis of p-JNK, p-p38, p-ERK and p-AKT expression.** MOVAS cells seeded in 9-cm plate were treated with 3 mM HPi for 0-14 days, or cells were cultured with CPECs-CM and treated with or without 3 mM high Pi for 14 days. p-JNK, p-p38, p-ERK and p-AKT were examined by western blotting. Bars with different letters indicate the significance at P<0.05 level.
